# CRISPR/Cas9-mediated one step bi-allelic change of genomic DNA in iPSCs and human RPE cells *in vitro* with dual antibiotic selection

**DOI:** 10.1038/s41598-018-36740-2

**Published:** 2019-01-17

**Authors:** Wasu Supharattanasitthi, Emil Carlsson, Umar Sharif, Luminita Paraoan

**Affiliations:** 1 Department of Eye and Vision Science, Institute of Ageing and Chronic Disease, University of Liverpool, Liverpool, United Kingdom; 2 Department of Physiology, Faculty of Pharmacy, Mahidol University, Bangkok, Thailand

## Abstract

CRISPR/Cas9 causes double-stranded DNA breaks that can undergo DNA repair either via non-homologous end joining (NHEJ) or, in the presence of a template, homology-directed repair (HDR). HDR is typically used to insert a specific genetic modification into the genome but has low efficiency compared to NHEJ, which is lowered even further when trying to create a homozygous change. In this study we devised a novel approach for homozygous single base editing based on utilising simultaneously two donor DNA templates cloned in plasmids with different antibiotic resistant genes. The donor templates were co-transfected alongside the CRISPR/Cas9 machinery into cells and a double antibiotic selection was optimised and allowed the isolation of viable desired clones. We applied the method for obtaining isogenic cells homozygous for variant B cystatin C, a recessive risk factor for age-related macular degeneration and Alzheimer’s disease, in both induced Pluripotent Stem Cells (iPSCs) and a human RPE cell line. Bi-allelic gene edited clones were validated by sequencing, demonstrating that the double antibiotic templates approach worked efficiently for both iPSCs and human differentiated cells. We propose that this one step gene editing approach can be used to improve the specificity and frequency of introducing homozygous modifications in mammalian cells.

## Introduction

CRISPR/Cas9 is a powerful method for editing and introducing specific changes to the genome^1–3^. One of the most challenging applications of the technique is the introduction of bi-allelic changes, which is often necessary for either obtaining experimental models or for correction of disease-related recessive variants. Such editing of both alleles is required in case of retinal degenerative diseases, sickle cell disease, β-thalassemia, cystic fibrosis^4^ and various mutations related to human cancer^5,6^.

Two main repair mechanisms exist in cells after the occurrence of double-strand DNA breaks: non-homologous end joining (NHEJ) and homology-directed repair (HDR). Several recent studies have attempted to improve the efficiency of HDR-mediated gene correction or modification^7–10^. As the efficiency of HDR is low (1–10% of modified alleles), the cells resulting from Cas9-driven cleavage events (that occur with higher efficiency) most often present NHEJ repaired alleles (wild-type or mutated) and only a very low proportion HDR-edited alleles^7^. Furthermore, if a desired change is required to occur on both alleles i.e. a homozygous change, the probability of achieving this via HDR is even lower and in most cases requires a double-step, sequential approach. Thus, the application of gene editing in relation to introducing a desired homozygous change in cells could greatly benefit from developing efficient experimental approaches that are less time consuming and more specific.

Here we describe a novel one step approach to modify both alleles with a desired change based on the use of two DNA donor templates with different antibiotic resistant genes. A bi-allelic nucleotide change in the CST3 gene was successfully introduced in both induced pluripotent stem cells (iPSCs) and a human retinal pigment epithelium cell line (ARPE-19) generating isogenic cells homozygous for variant B cystatin C, a recessive allele associated with increased risk of developing age-related macular degeneration and Alzheimer’s disease^11,12^. Based on our results, we expect that this approach can be applied to similar *in vitro* experiments employing CRISPR/Cas9 editing where efficiency is a concern or a limiting factor.

## Results

### CRISPR/Cas9-mediated gene editing of iPSCs

**Figure 1:**
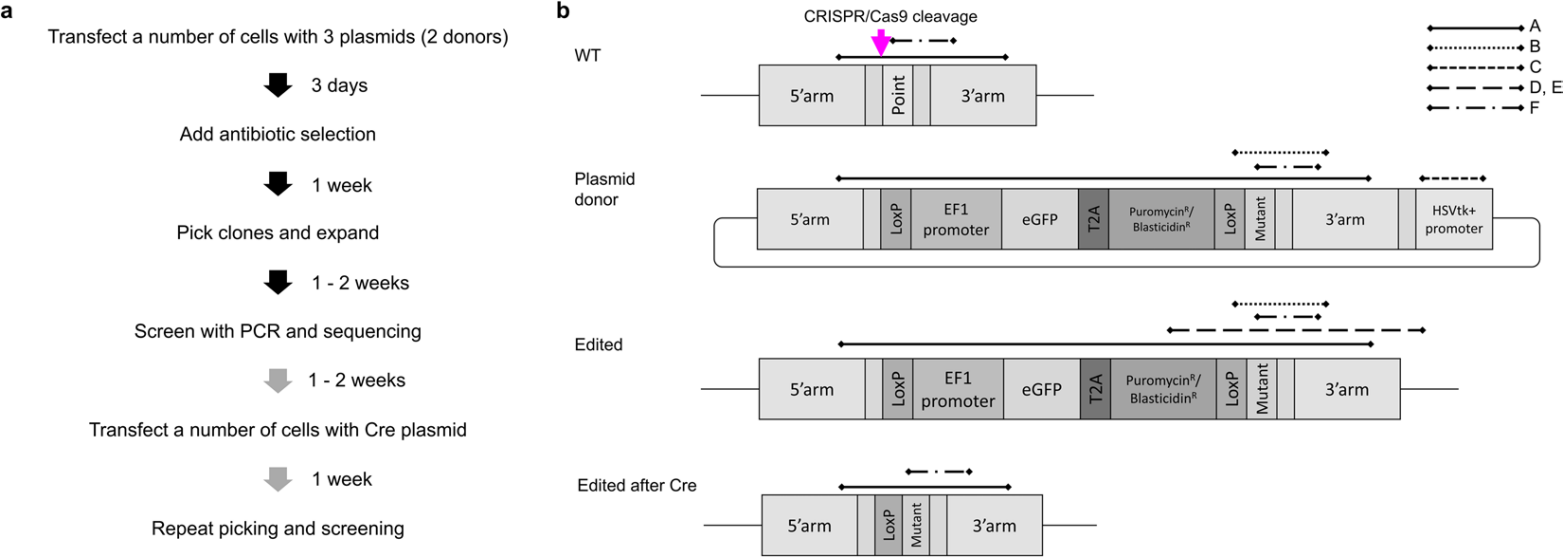
Overall processes and schematic diagrams of plasmid donor and genomic DNA in different stages of CRISPR/Cas9-mediated HDR. (**a**) Flow chart of the transfection and screening steps for isolation of gene edited clones. (**b**) Schematic illustrations of genomic sequences and donor plasmids. Four diagrams are shown: wild-type (WT), plasmid donor, edited sequence and edited sequence after removing a reporter cassette with Cre expression. CRISPR/Cas9 cleaves at the region shown with a magenta arrow on wild-type sequence. Lines drawn above diagrams indicate PCR fragments A-F as detailed in Table 1.

iPSCs are differentiable to multiple cell types, making them valuable for tissue-specific functional studies. However, they are also highly sensitive and prone to spontaneously differentiate during routine culture if care is not taken. We employed CRISPR/Cas9 methodology in order to introduce a bi-allelic mutation in the CST3 gene, which encodes cystatin C. Recommended protocols for establishing bi-allelic mutations typically involve insertion of a selection cassette into the target genome, followed by manual evaluation of individual clones to segregate between mono- and bi-allelic edits. Following multiple unsuccessful attempts to generate a bi-allelic mutation in the CST3 gene through the insertion of a puromycin-based selection cassette (58 clones screened, 0 with bi-allelic change), we devised a modified strategy for gene editing (Fig. 1a) using two separate donor plasmids (Fig. 1b). The only difference between the two donor plasmids is the inserted gene used for antibiotic selection, one using puromycin and the other blasticidin. By applying dual antibiotic selection after gene editing, only cells that have incorporated both genes for antibiotic resistance should in theory survive, meaning only bi-allelic edits should be present.

**Figure 2:**
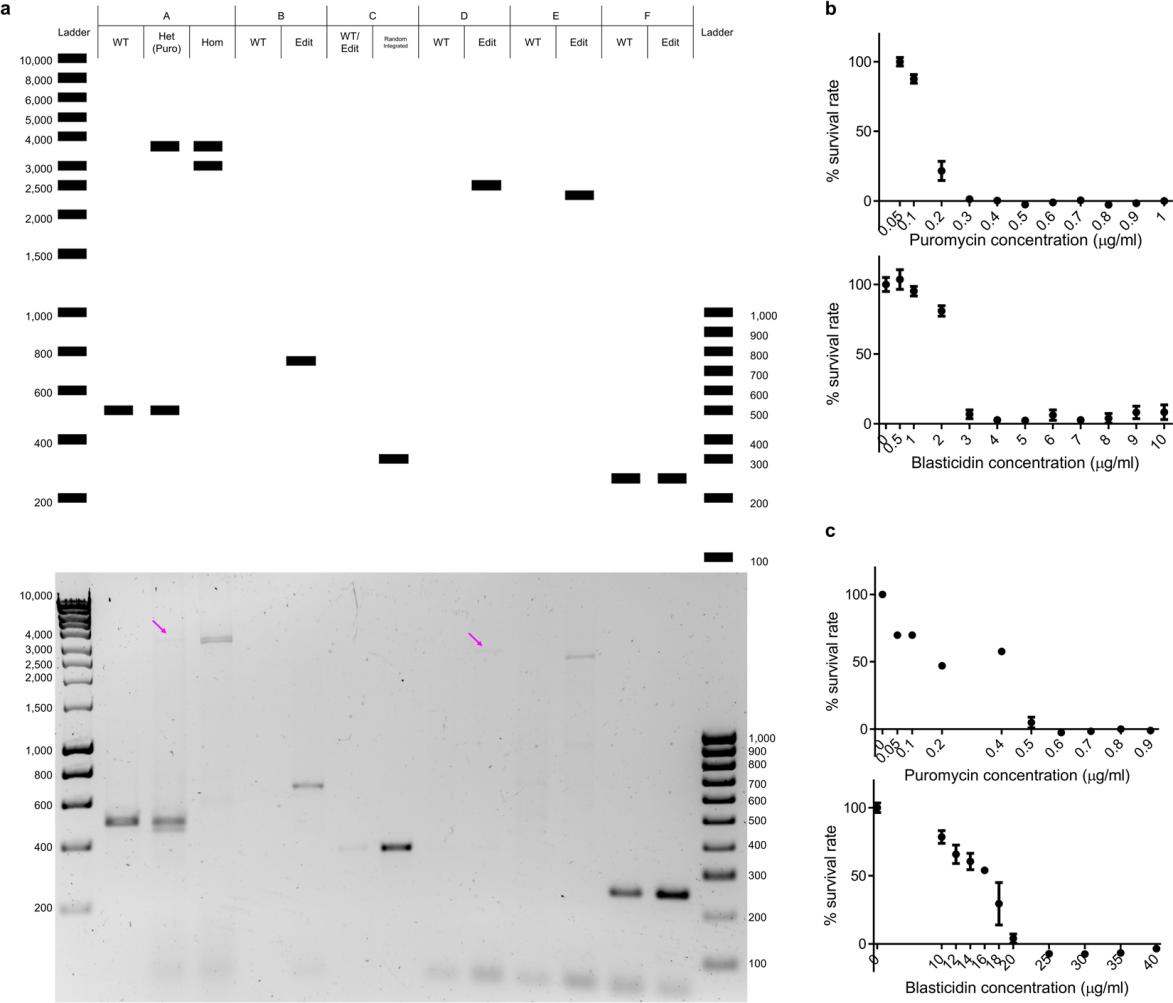
PCR screening of iPSCs gene edited with dual antibiotic selection. (**a**) PCR products were analysed by gel electrophoresis and referred to in relation to the different sources of templates: wild-type (WT), heterozygous changed (Het) with puromycin donor template (Puro), homozygous changed (Hom), CRISPR/Cas9 mediated HDR (Edit) and random plasmid integrated. Magenta arrows highlight faint bands in agarose gel. Sizes of the predicted PCR products (indicated in the top diagram) were confirmed by electrophoresis (full gel shown at the bottom). The resulting unique patterns could be used to differentiate homozygous gene edits from WT and heterozygous edits. (**b**) Antibiotic kill curves performed with iPSCs. Cells were grown with increasing concentrations of puromycin (top) and blasticidin (bottom) for 7 days. Endpoint survival was analysed by MTT assay. Means and SEM are shown in curves (**c**) Antibiotic kill curves performed with ARPE-19 cells. Means and SEM are shown in curves.

Electroporation of iPSCs was performed with the Neon electroporation kit. Although the manufacturer has made available protocols for electroporation of multiple established cell lines, no iPSC-specific protocol was available at the time of this study. We therefore initially used electroporation with a plasmid encoding eGFP with various parameters to optimise transfection conditions. As iPSCs are fragile and a large proportion of dead cells could be seen following electroporation under most of the conditions evaluated, we chose to use the lowest voltage which still resulted in clear eGFP expression. Following electroporation of iPSCs with both donor plasmids, together with the CRISPR/Cas9 plasmid, 12 clones could successfully be expanded and had their genomic DNA extracted and analysed. Specific rounds of PCR were applied to characterise clones in different regions on plasmids and genomic DNA (Fig. 1b) and provided a unique pattern for each pair of primers (Fig. 2a). One of these clones showed the expected pattern of PCR products for a successful bi-allelic edit, comprising 2 large bands with the A primers, single bands with B, D, and E primers and the absence of a band with the C primers (Fig. 2a). To ensure optimal concentrations of antibiotic selection, kill-curves were first established for both iPSCs (Fig. 2b) and ARPE-19 cells (Fig. 2c). As expected, iPSCs were found to be far more sensitive to both puromycin and blasticidin, with effectively no surviving cells at concentrations of 0.3 µg/ml and 4 µg/ml, respectively.

**Figure 3:**
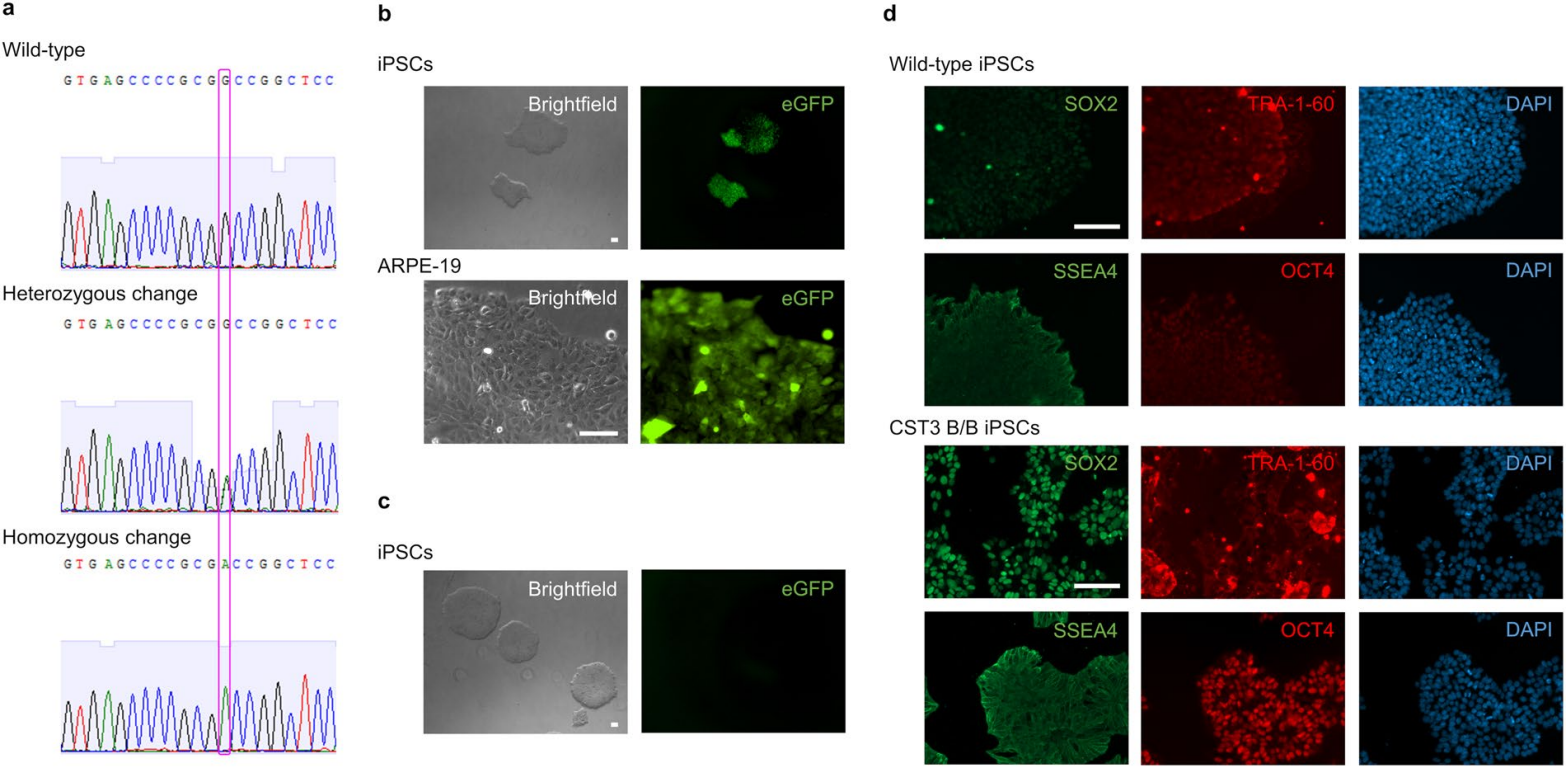
Verification by sequencing of gene editing and retention of iPSC pluripotency. (**a**) Sanger sequencing of genomic DNA extracted from gene edited iPSCs was used to identify clones harbouring hetero- or homozygous gene edits. The magenta box highlights the points of mutation. (**b**) Confirmation of successful gene editing via fluorescence microscopy. Clones viewed under fluorescence microscope after transfection displayed eGFP expression. Both iPSCs (top) and ARPE-19 cells (bottom) are shown. Scale bars 100 µm (**c**) Successful removal of selection cassette. iPS clone viewed under fluorescence microscope after removal of insertion cassette through Cre recombinase expression shows loss of eGFP expression. Images were captured using identical microscope settings as used for images shown in panel b. Scale bar 100 µm (**d**) Gene edited iPSCs are still pluripotent. PSC pluripotency staining of wild-type iPSCs (top) and the iPS clone after removal of insertion cassette (bottom) show continued expression of the four markers SOX2, TRA-1-60, SSEA4, and OCT4 following gene editing. Scale bars 100 µm.

To analyse whether the CST3 gene had been edited as predicted, the PCR product resulting from amplification using primer pair F was subjected to Sanger sequencing, which confirmed the homozygous G73A nucleotide variant in the CST3 gene (Fig. 3a). As an additional control, and to test whether our strategy of using two different insertion cassettes had worked as expected, amplicons from PCR reactions using D and E primers, which amplify specific regions for each antibiotic resistant gene, were used as templates to re-amplify the region spanning the intended mutation with F primers. Sanger sequencing confirmed that the mutation was present in both of the antibiotic-based amplicons (data not shown).

### Removal of insertion cassette from gene edited iPSCs

Gene edited iPSCs were expanded and subjected to at least one freeze/thaw cycle in liquid nitrogen to ensure the robustness of the cells, after which the inserted cassettes were removed via Cre-recombinase expression. As shown in Fig. 1b, two LoxP sites are flanking the inserted selection marker regions, which make these regions targets for excision. Following electroporation using the pCMV-CRE plasmid, cells were subjected to the same methods and controls as during the editing processes. As the selection cassettes contain in addition to genes for antibiotic resistance also the eGFP gene, successful gene editing could be manually evaluated through fluorescence microscopy (Fig. 3b). Consequently, removal of the insertion cassettes also resulted in a loss of eGFP expression (Fig. 3c).

Although confirmation of successful editing via sequencing is enough to demonstrate the viability of the protocol from a technical standpoint, the usefulness in this specific example was still dependent on the iPSCs retaining their pluripotent state throughout the process. To analyse whether this was the case, PSC pluripotency staining was used to compare wild-type iPSCs and the iPS clone after removal of insertion cassette. As shown in Fig. 3d, immunostaining against pluripotency markers SOX2, TRA-1–60, SSEA4, and OCT4 clearly showed expression all four markers in the edited cells.

### CRISPR/Cas9-mediated gene editing of ARPE-19 cells

To demonstrate a broader applicability of this editing strategy, we also used it to edit the genome of the human cell line ARPE-19. This is a commonly used and robust cell line of RPE cells, and given that cystatin C is highly expressed by these cells a modified line homozygous for variant B expression may prove useful in downstream applications. The editing method applied to ARPE-19 cells was identical to that used for iPSCs with the exceptions of electric parameters during electroporation, cell maintenance and antibiotic concentrations used during the selection process. In total, seven surviving clones were obtained and expanded, and two clones showed the expected PCR pattern and Sanger sequencing results from a homozygous edit, just as the corrected iPSC clone (Fig. 3b).

## Discussion

CRISPR/Cas9 system provides the opportunity to correct erroneous genomic DNA in many organisms^4,13,14^. However, one of the most difficult aspects of CRISPR/Cas9 technology is the low efficiency of repairing double-strand breaks via HDR when a donor template is provided. The resulting population of cells on which CRISPR/Cas9 has been applied will contain mostly wild-type and NHEJ repaired alleles with a low proportion of HDR edited alleles. In addition, if the purpose of the HDR is to introduce a homozygous change, the chance of obtaining this and related efficiency are further lowered. Improved efficiency of HDR homozygous change is thus required both for generation of experimental models of diseases which are caused by a homozygous mutation and for enabling potential new treatment avenues.

In this study, a methodological approach has been devised and applied in which the gRNA, Cas9 nuclease and two identical donor DNA templates (except for difference in antibiotic selection) were delivered into iPSCs resulting in radically increasing the efficiency of introducing a homozygous change in *in vitro* cell cultures. When using a double antibiotic approach, the mechanism of action of both antibiotics should not interfere with the interaction of DNA or RNA processes that may inhibit CRISPR/Cas9 activity. Both antibiotics must also be compatible and not show any signs of drug-drug interaction – a condition that is met by the combination of puromycin and blasticidin used in this study^15–17^.

By introducing two DNA templates with different antibiotic resistance genes, clones surviving the selection process should theoretically contain the desired change in both alleles, i.e. one allele containing the puromycin resistance template whereas the second allele containing the blasticidin resistance template. The use of both antibiotic resistant genes, which are slightly different in term of sequence length, also helped with the PCR screening of these clones (Fig. 2a) due to the different sizes of respective PCR amplicons. When this method was applied to iPSCs in this study, twelve clones were selected from which one showed the desired change as determined by PCR screening and Sanger sequencing (efficiency of 8.3%). This approach was also applied to ARPE-19 cells and here an efficiency of 28.6% was achieved (Table S1). A recent study demonstrated an efficiency of around 8% for a homozygous change in human embryonic stem cells for the gene of interest when using a standard one antibiotic approach^18^.

Our study provided a clear proof of principle and initial optimisations for the use of a double antibiotic approach in CRISPR/Cas9-mediated homozygous single base gene editing. Although the overall efficiency was improved compared to the use of one antibiotic approach, this can be further improved by altering certain details of the design used. One way of improving the design is to bring the Cas9 binding site closer to the point of interest allowing the DNA break and subsequent digestion before the HDR event to occur more efficiently. In addition to this, the HDR efficiency can be improved by using single-stranded DNA (ssDNA) instead of the double-stranded donor template since the use of ssDNA was shown to increase the HDR outcome and to decrease random integration^19^. Furthermore, to help reduce off-target effects, the use of Cas9 nickase with two gRNAs which cover the point of interest can be considered.

Improvement of the delivery of the CRISPR/Cas9 machinery into cells can also be considered. Of particular value for this study proved to be the cell-specific optimisation of the electric parameters of electroporation as the use of electroporation may result in DNA damage as well as cell death. Before addition of CRISPR/Cas9 machinery into cells, the transfection of eGFP plasmid was used to measure and optimise the minimum levels of voltage, pulse width and number of pulses needed to observe eGFP expression inside the cell. These values were then applied to the actual experiments to deliver CRISPR/Cas9 and donor templates to the cells. However, it is not clear if the transfection efficiency for the plasmids used during gene editing differs from that of the eGFP plasmid. If electroporation is not a viable option for a study, we predict that other ways of plasmid delivery such as viral vectors, nanoparticles or nucleic acid injection could be applied alongside the double antibiotic approach used in this study^3,20,21^.

As the dual antibiotic approach is effective and simple in principle, it is applicable to many different cell types. The method has an efficient outcome and is less time consuming compared to standard approaches making it a very appealing way of introducing homozygous genetic alterations in cells. The ability to efficiently introduce homozygous changes will open up avenues for the study of diseases at a molecular level through the development of *in vitro* disease-in-a-dish experimental models. These models will allow better understanding of how variant protein products contribute to disease through elucidating mechanisms at the molecular level, thus eventually giving rise to an opportunity for therapeutic advancements.

## Methods

### Cell culture

Human iPSCs were supplied by The Human Induced Pluripotent Stem Cells Initiative (HipSci) and maintained according to general guidelines for handling human iPSCs (document number M211.20160208.v1). Briefly, 6-well plates (catalogue number 657160, Greiner Bio-One Ltd.) were coated with vitronectin (catalogue number A14700, Life Technologies Ltd.) for 1 hour before seeding iPSCs. Cells were maintained in Essential 8™ Medium (catalogue number A1517001, Life Technologies Ltd.) at 37 °C and 5% CO_2_. Daily feeding continued until 80% confluency. iPSCs were passaged by washing twice with Dulbecco’s phosphate buffered saline (DPBS, catalogue number 14190144, Life Technologies Ltd.) then harvested with an EDTA solution (Versene, catalogue number 15040066, Life Technologies Ltd.). Cells were fed with Essential 8™ medium supplemented with 10 µM Rock inhibitor (catalogue number 72304, STEMCELL Technologies UK Ltd.) on the day after splitting and media was then changed to normal Essential 8™ Medium on the following day.

ARPE-19 cells (ATCC) were maintained in Dulbecco’s modified Eagle’s medium/nutrient mixture F-12 Ham (DMEM F12, catalogue number 8437, Sigma-Aldrich Company Ltd.) containing 10% fetal bovine serum (catalogue number F7524, Sigma-Aldrich Company Ltd.) at 37 °C and 5% CO_2_. Cells were routinely passaged by washing twice with Dulbecco’s phosphate buffered saline (DPBS, catalogue number D8537, Sigma-Aldrich Company Ltd.) and dislodged using 1 × Trypsin-EDTA solution (catalogue number T4174, Sigma-Aldrich Company Ltd.) when they reached 80% confluence.

### Plasmid template and guide RNA

Plasmid donor templates and guide RNA (gRNA) were custom-designed with sequences verified by System Biosciences (SBI). The customised donor template contained a 5′ homology arm, LoxP site, EF1 promoter, reporter genes (eGFP with puromycin resistance gene), a second LoxP site, point of mutation, 3′ homology arm and another reporter gene (extra reporter: PGK promoter with human herpes simplex virus thymidine kinase type 1 gene (HSVtk)). The customised donor template was modified with restriction enzymes from Roche Diagnostics Ltd., Phusion high-fidelity PCR kit (catalogue number F553S, Life Technologies Ltd.) and Mighty Mix DNA Ligation Kit (catalogue number 6023, Takara Bio Europe SAS) to change the puromycin resistance gene to a blasticidin resistance gene amplified from the PSF-CMV-BLAST plasmid (catalogue number OGS588, Sigma-Aldrich Company Ltd.) to create the second donor DNA plasmid. All cloning steps were performed according to manufacturers’ protocols.

### Establishment of antibiotic selection conditions

ARPE-19 cells and iPSCs were cultured with a range of puromycin (catalogue number ant-pr-1, InvivoGen) or blasticidin (catalogue number ant-bl-1, InvivoGen) concentration for 7 days. Vybrant® MTT cell proliferation assay kit (catalogue number V13154, Life Technologies Ltd.) was then used to measure survival rates at the end point, according to manufacturer’s instructions. The lowest concentrations of antibiotics that killed all cells were considered optimal and used in subsequent selection experiments.

### Electroporation

ARPE-19 cells and iPSCs were transfected using the Neon® transfection system (Life Technologies Ltd.) in accordance with manufacturer’s instructions. Briefly, for ARPE-19 cells, 5 × 10^6^ cells were harvested by centrifugation at 300 *g* for 5 min, washed with DPBS and resuspended in 100 µL buffer R. 5 µg of each donor plasmid, together with 5 µg of Cas9 plasmid, were added to the suspension and electroporation was performed using parameters 1350 V, 20 ms, 2 pulses, after which cells were grown in DMEM F12 medium. For iPSCs, cells were fed with Essential 8™ medium supplemented with 10 µM Rock inhibitor 30 minutes before electroporation. After harvesting with StemPro® Accutase® cell dissociation reagent (catalogue number A1110501, Life Technologies Ltd.), 3 × 10^6^ cells were collected and resuspended in 100 µl buffer R. 10 µg of donor plasmid (two preparations), together with 5 µg of Cas9 plasmid, were added to the suspension and electroporation was performed using parameters 1400 V, 20 ms, 1 pulse, after which cells were grown in NutriStem® (catalogue number: 01-0005, ReproCELL Europe Ltd.) medium.

### Antibiotic selection and extraction of genomic DNA

Following electroporation, cells were seeded into 10 cm dishes (catalogue number 664160, Greiner Bio-One Ltd.) and cultured at 37 °C and 5% CO_2_. 3 days post-transfection, dual antibiotic selection pressure was applied in accordance with previously established kill curve performance and cells were cultured for a further 8 days. For ARPE-19, 0.6 µg/ml puromycin and 25 µg/ml blasticidin were used while 0.3 µg/ml puromycin and 4 µg/ml blasticidin were used for iPSCs. Cells were then washed twice with DPBS, and individual clones were picked with a loop (catalogue number 731165, Greiner Bio-One Ltd.) and moved into separate wells on a 24-well plate (catalogue number 662160, Greiner Bio-One Ltd.). Clones were expanded until near confluence, after which genomic DNA was harvested with DNeasy blood & tissue kits (catalogue number 69506, QIAGEN Ltd.).

### PCR screening and Sanger sequencing

Extracted genomic DNA was used as template for PCR screening using primers (Eurogentec Ltd. or Life Technologies Ltd) shown in Table 1. Individual reactions were set up with *Taq* PCR Master Mix Kit (catalogue number 201445, QIAGEN Ltd.) with the addition of 5% dimethyl sulfoxide (catalogue number D8418, Sigma-Aldrich Company Ltd.) and thermocycling was performed using a Veriti® 96-Well Fast Thermal Cycler (Life Technologies Ltd.). Selected PCR products were sequenced by Source BioScience or DNA Sequencing and Services, University of Dundee.

**Table 1:** Primers, binding site, function and referable PCR product size.

Primer	Binding site		Function	PCR product size		
	Forward	Reverse		Wild type	heterozygous	homozygous
A	5′ homology arm	3′ homology arm	Classify wild type and edited	+	+ and ++	++
B	Inserted part	3′ homology arm	Prove inserted	−	+	+
C	Extra reporter	Extra reporter	Prove random integrated	−	−	−
D	Puromycin resistant gene	Downstream 3′ homology arm genomic DNA	Prove the edited allele with puromycin resistant gene at correct region	−	+	+
E	Blasticidin resistant gene	Downstream 3′ homology arm genomic DNA	Prove the edited allele with blasticidin resistant gene at correct region	−	+	+
F	Before point of interest	After point of interest	Prepare for sequencing	+	+	+

−: no product, +: positive product, ++: positive product of a larger size.

### Removal of insertion cassette and verification of gene editing

Clones were electroporated using the same protocol described above with Cre recombinase expression plasmid (pCMV-CRE, catalogue number CRE100A-1, Cambridge Bioscience Ltd.) and cultured for 5–7 days. Individual clones were picked and analysed by PCR screening and Sanger sequencing as described above to verify successful removal of the inserted cassette.

### Immunofluorescence

The iPSC staining of selected pluripotency markers was performed using a PSC 4-marker immunocytochemistry kit (catalogue number A24881, Life Technologies). 10,000 cells were seeded per well in a 96-well plate and cultured for 48 hours, followed by fixation in 4% formaldehyde in DPBS for 15 minutes, permeabilisation with 1% saponin in DPBS for 15 minutes, blocking with 3% BSA in DPBS for 30 minutes, and immunostaining with indicated antibodies diluted in blocking buffer for 3 hours. Primary antibodies used were mouse anti-SSEA4 (1:100), rabbit anti-OCT4 (1:100), rat anti-SOX2 (1:100), and mouse anti-TRA-1–60 (1:100). Cells were then washed three times with DPBS and stained with secondary fluorescent antibodies for 1 hour. Secondary antibodies used were Alexa Fluor 488 goat anti-mouse IgG3 (1:250), Alexa Fluor 594 donkey anti-rabbit IgG (1:250), Alexa Fluor 488 donkey anti-rat IgG (1:250), and Alexa Fluor 594 goat anti-mouse IgM (1:250). Cells were finally washed three times with DPBS, stained with DAPI and imaged using a Zeiss Apotome inverted fluorescence microscope.

## Electronic supplementary material

Supplementary information
