# Supplementary material for: CRISPR/Cas9-mediated one step bi-allelic change of genomic DNA in iPSCs and human RPE cells in vitro with dual antibiotic selection
Source: Sci Rep. 2019 Jan 17;9:174. doi: 10.1038/s41598-018-36740-2 (PMC6336765; doi:10.1038/s41598-018-36740-2)
Supplement: Supplementary file 1 — Supplementary information [file 41598_2018_36740_MOESM1_ESM.pdf]

Supplementary information

CRISPR/Cas9-mediated one step bi-allelic change of genomic DNA in iPSCs and human RPE cells *in vitro* with dual antibiotic selection

Wasu Supharattanasitthi[1, 2], Emil Carlsson[1], Umar Sharif[1], Luminita Paraoan[1,*]

[1]Department of Eye and Vision Science, Institute of Ageing and Chronic Disease, University of Liverpool, Liverpool, United Kingdom;

[2]Department of Physiology, Faculty of Pharmacy, Mahidol University, Bangkok, Thailand

*Corresponding author: Luminita Paraoan, PhD

Department of Eye and Vision Science, Institute of Ageing and Chronic Disease, University of Liverpool, William Duncan Building, 6 West Derby Street, Liverpool L7 8TX, United Kingdom

Tel.: +44 151 794 9038

E-mail: lparaoan@liverpool.ac.uk

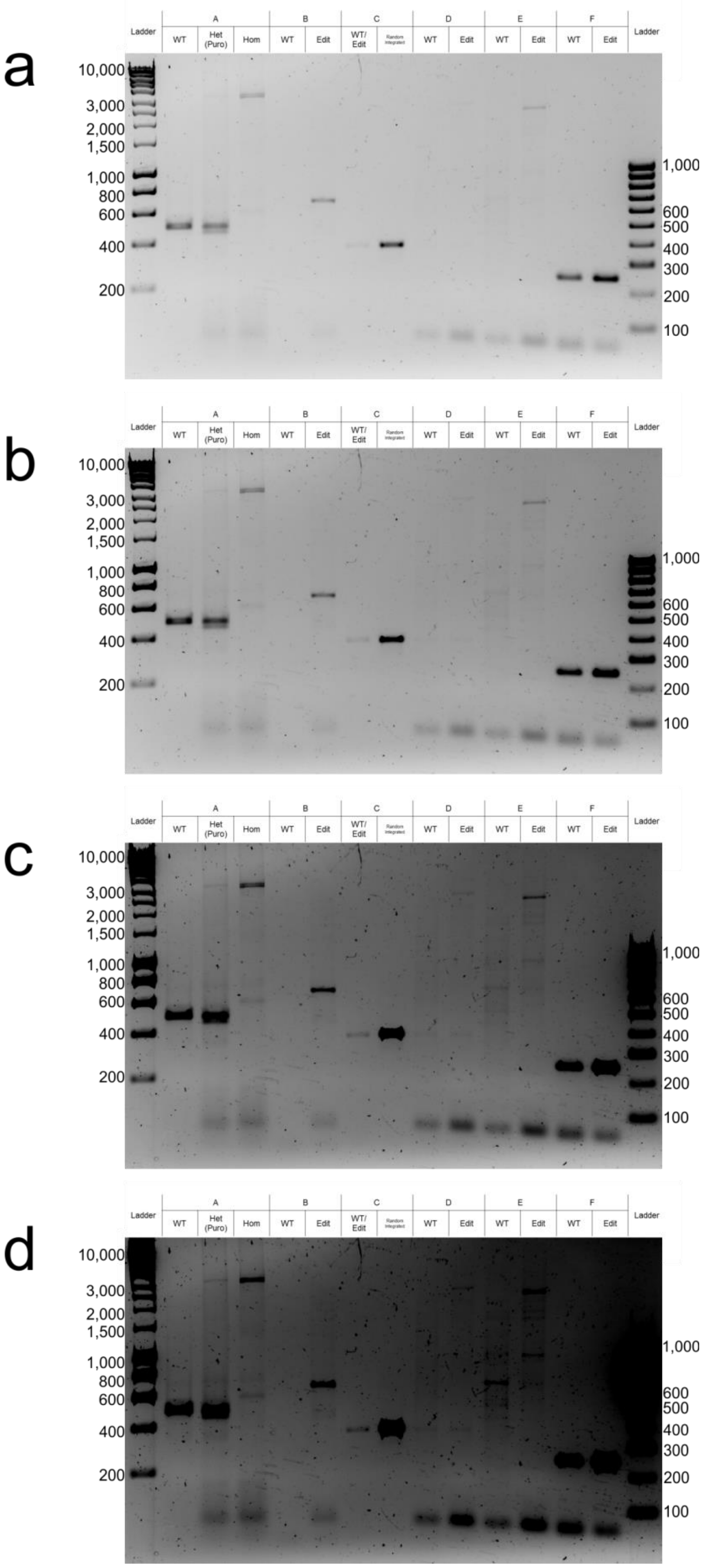

Supplementary Fig. 1. Images of full length agarose gel shown in Figure 2 with multiple exposures. PCR products were loaded on a single agarose gel and analysed by electrophoresis with the full length gel exposed for 0.643 s **(a)**, 1.000 s **(b)**, 1.500 s **(c)**, and 2.000 s **(d)**.

Supplementary Table 1. Efficiency of bi-allelic change in iPSCs and ARPE-19 cells.

	Single antibiotic approach	Dual antibiotic approach
iPSC	0% (58 clones screened)	8.3% (12 clones screened)
ARPE-19	N/A	28.6% (7 clones screened)
